# Routine pre-employment echocardiography assessment in young adults: cost and benefits

**DOI:** 10.1186/s43044-020-00131-8

**Published:** 2021-01-06

**Authors:** Ahmed Gaafar, Asmaa Gaafar

**Affiliations:** 1 Cardiology Department, Faculty of Medicine, Helwan University, Cairo, Egypt; 2 Psychology Department, Faculty of Humanities, Al-Azhar University, Cairo, Egypt

**Keywords:** Echocardiography, Pre-employment assessment psychological stress, Money cost

## Abstract

**Background:**

Conventional echocardiography is a safe, available, and accurate tool for cardiac structural and functional evaluation, but it should not cancel clinical assessment and history tacking, and indeed both are complementary. A pre-employment assessment is important for employees and community safety and suitability for a specific work requirement.

**Results:**

Aiming to assess the value of routine pre-employment echocardiography for the detection of cardiac abnormalities, we examined seven hundred ninety-five persons who were routinely referred to us for pre-employment conventional echocardiography. Only 9 persons had structural cardiac abnormalities (1.3%) and distributed as follows: two had bicuspid aortic valve with isolated aortic regurgitation, one of them had mild AR, and the other had moderate AR. Two cases had mitral valve prolapse, one of them had trivial MR, while the other had a flail anterior leaflet with severe MR. One patient had atrial septal defect 1.5 cm with mild pulmonary hypertension and right-sided chambers dilatation. One patient had dextrocardia (situs inversus totalis) without other cardiac problems. One had moderate pulmonary hypertension and modest right-sided chambers dilation. Two patients had left ventricular hypertrophy. Surprisingly, we did not detect rheumatic valvular heart disease. The money cost of echocardiography tests for those 795 persons was 198,750 Egyptian pounds (LE); their transportation cost was about 19.800LE. The total group time cost of the tests was 265 h, total time lost at the waiting room was 1590 h, total transportation time lose was 2385 h, so the total time cost was about 4000 h. Using psychological stress questionnaire, 33 participants (4.2%) had results suggestive of a low sense of psychological pressure due to echocardiography examination, 221 participants (27.8%) had results suggestive of a moderate feeling of stress, while 541 participants (68%) had results suggestive of a high sense of stress.

**Conclusion:**

We recommend against routine echocardiography for cardiac assessment in pre-employment assessment and to do it only for persons with abnormal clinical or ECG findings.

## Background

Assessment of fitness for work definition is the assessment of a worker’s capacity to work without risk to their own or others’ health and safety. The medical examination offers several purposes for the benefit of the employer and the employee. It is done to ensure that the prospective employee has no “serious medical condition” and he is “physically and mentally” suitable for the job offered. But in some jobs, the demands of the work requirements that the prospective employee should meet the minimum required medical standards to qualify for the appointment [1].

Despite the argument about the practical benefits of screening tests, there is a true desire to screen populations under cardiovascular risk; the screening aims to detect underlying diseases or abnormalities and subsequently reduce morbidity and mortality, but unfortunately, there is no agreement about the most proper and reliable test which can effectively detect the disease or abnormalities [2].

The leading cause of sudden cardiac death is well known which is a cardiovascular disease as silent ischemic heart disease, lethal arrhythmias, and myocardial diseases as hypertrophic cardiomyopathy, so early detection, diagnosis, and proper management of silent cardiovascular diseases are of great importance [3].

The AHA guidelines recommended a fourteen elements comprehensive medical questionnaire of the personal and family history and physical examination as a guide for examiners to use it when they perform the pre-participation examinations for adolescent athletes (Table 1) [4].

**Table 1:** The 14-element AHA recommendation for pre-participation cardiovascular screening of competitive athletes

Medical history (parental verification recommended for high school and middle school athletes)	
Personal history	1.Exertional chest pain/discomfort
	2.Exertional syncope or near-syncope
	3.Excessive exertional and unexplained fatigue/fatigue associated with exercise
	4.Prior recognition of a heart murmur
	5.Elevated systemic blood pressure
	6.Prior restriction from participation in sports
	7.Prior testing for the heart ordered by a physician
Family history	8.Premature death–sudden and unexpected before age 50 year due to heart disease, in one or more relatives
	9.Disability from heart disease in a close relative < 50 years old
	10.Specific knowledge of certain cardiac conditions in family members: hypertrophic or dilated cardiomyopathy, long QT syndrome or other ion channelopathies, Marfan syndrome, or clinically important arrhythmias
Physical exam	
11. Heart Murmur—exam supine and standing or with Valsalva, specifically to identify murmurs of dynamic L ventricular outflow tract obstruction	
12. Femoral pulses to exclude aortic stenosis	
13. Physical stigmata of Marfan syndrome	
14. Brachial artery blood pressure (sitting, preferably taken in both arms)	

Positive/abnormal screen warrants further evaluation and 12-lead EKG

AHA does Not currently recommend routine 12-lead ECG

Also, the US military recommended mandatory screening for cardiovascular disease military recruits using screening questionnaires and physical examination, but ECG is only performed in selective cases guided by history, symptoms, or physical data, and as a routine for aviation duty and in soldiers > 40 years old [2].

Nistri et al. used data from 34,910 persons ≥ 18 years old at the military screening program from 1992 to 1996 based on history-taking, physical examination, and routine ECG, and they found 8% had abnormalities and were referred for echocardiographic examination; echocardiography revealed that 0.7% of them had a new HCM diagnosis [5].

Unfortunately, personal history, family history, and physical examination have low sensitivity to detect many diseases and abnormalities that can cause sudden cardiac death [6].

A retrospective study of suddenly died competitive athletes revealed only < 5% of them had a confirmed cardiovascular diagnosis after screening with standard history and physical examination [7].

For example, by ECG only you can detect ion channelopathies and Wolff Parkinson-White syndrome. Many structural cardiac diseases which may lead to SCD, symptoms, family history of heart disease, and clinical signs are infrequent findings. For example in HCM, a systolic murmur is audible at rest in 25% of patients and with dynamic auscultation, it is audible in an additional 50% [8].

The British Army screening system although they described transthoracic echocardiography as relatively cheap, fast, and accessible in cardiac screening for potential recruits, they considered the need for professionals specialized in echocardiography increases the cost of the screening system and reduces the number of patients that can be investigated. Their final recommendation was to perform routine echocardiography only when the cost is a less important factor and the numbers of participants are low, as screening professional football players [9]. So, the International Federation of Association Football (FIFA), the International Cycling Union (UCI), and the U.S. National Basketball Association (NBA) perform routine echocardiography in the primary screening tests in asymptomatic athletes [10].

The pre-employment screening test is just one factor of many factors used to judge the suitability of someone for the job. We cannot ignore the psychological stress induced by the pre-employment test. “Fear of failure to individuals who tie their self-worth to the outcome of pre-employment assessment” is particularly true for prospective employees, as the evaluative and competitive nature of the process of job application often causes feelings of anxiety, frustration, and distress [11].

### Aim of the study

We aimed to assess the usefulness and cost of using echocardiography as routine practice in pre-employment assessment.

## Methods

This a prospective survey study included 795 young persons (772 males, 23 females), with an age range between 20 and 31 years referred for routine echocardiography assessment as a screening test before employment in a variety of manual work jobs on railway lines and maintenance workshops of Egypt Railways, at the period between 1/2020 and 4/2020.

All persons were subjected to full history tacking using the AHA questionnaire for the cardiovascular screening of young athletes before competitive sports (Table 1) [12].

Also, a full cardiac examination was done for all subjects with special concerns for BP, cardiac murmurs, and dynamic auscultation to avoid missing LVOT obstruction, also a routine assessment of peripheral and femoral pulsations to detect aortic coarctation.

Routine 12-lead ECG was obtained to assess electrical abnormalities, myocardial ischemia, or chambers abnormalities. We did ECG at speed 25 mm/s with normal standardization (10 mm/1 mV) using automated mode.

Using the recommendations of last published American Society of Echocardiography [13], conventional transthoracic two-dimensional echocardiography using Philips HD11 (Philips Medical Systems; Eindhoven, Netherland) machine was done routinely; we used basic echocardiographic views (left parasternal long and short axis, apical 4, apical 2, and apical 3 chambers views, subcostal and suprasternal views) while the subjects at a left lateral position and ECG gated to assess chambers sizes, myocardial mass, and LV wall thickness, ventricular systolic function (by M-Mode, and area length method when needed) and diastolic function (using pulsed-wave Doppler and pulsed wave tissue Doppler to calculate E/A, E’/A’ and E/E’), valvular morphologies and functions, great vessels diameters, pericardium, and finally to exclude congenital abnormalities.

The echocardiography test cost and transportation cost of each person were obtained to be used for the calculation of total cost.

The time of echocardiography exam, transportation time, and waiting time for each subject was recorded.

As regards our knowledge, we did not find a questionnaire focusing on psychological stress induced by pe-employment medical assessment, so we developed the Pre-employment psychological Stress Scale (PSS) to assess and escalating the psychological stress induced by pre-employment medical assessment (Table 2).

**Table 2:** Psychological stress questionnaire

	Item	Agree	Rather agree	Disagree
1	I feel confident in my efficiency.			
2	Sometimes I worry about my health.			
3	My thinking about the consequences of a medical examination makes me nervous.			
4	I see that the administrative decision to accept the job is unfair.			
5	I am afraid not to secure an adequate physical level if I am not employed.			
6	I have negative thoughts such as expectation of rejection or illness			
7	I am scared of refusing to hire me.			
8	I'm afraid my situation will get worse if I don’t get the job.			
9	I feel that the medical examination of the job is annoying and disturbing.			
10	I am afraid of something that threatens my job.			
11	I am afraid that the result of the medical examination will affect my private life.			
12	I am concerned to think that I am seriously ill.			
13	I fear my relationship with my family will get worse if I don't get a job.			
14	I see the medical examination as a threat to some.			
15	I am afraid not to get another job if I am refused.			

The total score of the questionnaire ranges between (15) minimum score, and (45) degree maximum, where it indicates:

• The range of degrees (15–25) suggested a low level of psychological stress

• The range of degrees (26–36) suggested a medium level of psychological stress

• The range of degrees (37–45) suggested a high level of psychological stress

The questionnaire was used to assess the person’s psychological stress due to referral for echocardiography assessment. It contains (15) items, and suggested three alternatives for the answer (agree, rather agree, disagree), Key correction (3, 2, 1).

* The total score of the questionnaire ranges between 15 and 45°, where it indicates:

- The range of degrees (15–25) suggested a low level of psychological stress.
- The range of degrees (26–36) suggested a medium level of psychological stress.
- The range of degrees (37–45) suggested a high level of psychological stress.

An exploratory study was conducted on an experimental sample representing the original study sample for the following aims:

Determining the psychometric properties of the scale used in the study, so the authors can carry out the basic study through a scale of enough validity and stability.

The exploratory sample represents the individuals of the basic sample in all its specifications (gender and age distributions) to standardize study scale on them through validity and stability inappropriate ways. The exploratory sample consisted of (80) individuals, males and females (78 males 79.5% and 2 females 2.5%), whose ages ranged in the age group (24–33) years so that the individuals of the basic sample were represented in all their characteristics.

### Statistical methodology

Data were collected, revised, coded, and entered into the statistical package for social science (SPSS) version 21. Qualitative (categorical) data were presented as numbers and percentages; quantitative (continuous) data were presented as mean and standard deviations. The *p* values were considered significant if less than 0.05.

The test of the internal consistency of the questionnaire revealed that all 15 items are highly correlated with the total score. The Cronbach’s alpha for the inventory was (0.77) indicating a respectable degree of internal consistency. The alpha for the males was (0.78), and for females was (0.75). A re-test after 6 weeks showed a significant correlation (*r* = 0.64, *p* > 0.01) between test and re-test scores. This allows the authors to use the scale in the current study.

## Results

Aiming to assess the value of routine pre-employment echocardiography for the detection of cardiac abnormalities, we examined seven hundred ninety-five young persons who were routinely referred to us for pre-employment conventional echocardiography.

Mean heart rate of the studied subjects was 85.2 ± 9.8, (range, 63–120) and categorized them into three groups, group 1: HR of 63–80 b/m and included 233 subjects (29.3%); group 2: HR of 81–100 and included 417 subjects (52.5%); and group 3: HR of 101–120 b/m and included 145 subjects (18.2%).

Seven hundred seventy-four persons (97.4%) were male and 21 (2.6%) were female with a mean age was 25 ± 8.4 years old. Further, 193 (24%) persons were smokers; all of them were men. Seven (0.9%) persons had elevated BP at the examination; two of them were known to be hypertensive patients on medical treatment and the other five subjects had no history of hypertension and were referred to a hypertension clinic to complete their assessment. Two persons (0.25%) were diabetics; one was newly discovered and the other one was known type 1 DM on medical treatment (Table 3).

**Table 3:** Baseline demographic data of the whole study population

Demographic data	All patients
Count (%)	795 (100%)
Age (years)	
Mean ± SD	25 ± 8.4
Range	24–33
Gender	
Male	774 (97.4%)
Female	21 (2.6%)
Risk factors and history	
Elevated BP	7 (0.9%)
DM	2 (0.25%)
Smoking	193 (24%)
Family history of SCD	4 (0.5%)
Family history of premature IHD	3 (0.37%)
Syncope	2 (0.25%)

*BP* blood pressure, *DM* diabetes mellitus, *SCD* sudden cardiac death, *IHD* ischemic heart disease

By history, four (0.5%) subjects had a family history of SCD; only two of them occurred at a young age (undiagnosed) while the other two SCD cases occurred at age 65 and 69 (due to ACS); the two patients with a family history of SCD at a young age had normal ECG cardiac examination and normal echocardiography without a history of any syncopal attacks. Three (0.37%) subjects had a family history of premature IHD. Two (0.25%) persons had a history of single attack of syncope were diagnosed as neurogenic syncope (one of them syncopal attack occurred during hard work in hot weather and the other during prolonged fasting when he was in long-standing in a queue).

ECG assessment revealed 14 subjects (1.7%) had abnormal ECGs, three subjects had infrequent PVCs, two subjects had LVH, one subject had RBBB, one subject had dextrocardia pattern, two subjects had abnormal cardiac axis, four persons had non-specific T wave changes, and one subject had RVH (Table 4).

**Table 4:** Baseline clinical, ECG, echocardiographic, and laboratory data

Clinical, ECG, echocardiographic and laboratory data	All patients
**SBP (mmHg)**	
Mean ± SD	107.1 ± 18.0
Range	90–150
**DBP (mmHg)**	
Mean ± SD	87.5 ± 12.4
Range	60–100
**Pulse (beat/min)**	
Mean ± SD	85.2 ± 9.8
Range	63–120
**ECG**	
Normal	781 (98.3%)
Abnormal	14 (1.7%)
**Echocardiography**	
**EF (%)** mean ± SD (range)	62.9 ± 7.8 (54–76)
LVEDd (cm)	4.9 ± 0.81
**IVSd** (cm)	0.82 ± 0.21
PWd (cm)	0.79 ± 0.18
LA area (cm^2^)	17 ± 3.1
RVd (cm)	3.2 ± 1.1
TV max systolic velocity (m/s)	2.1 ± 4.7
MV E/E’ (range)	6.2 ± 3.1 (3.2–8.9)
**Lab** mean ± SD	
**Creatinine (mg/dL)**	0.8 ± 0.24
**Hb (gm/dl)**	11 ± 3.7
**TSH (IU/mL)**	2.7 ± 1.8

*SBP* systolic blood pressure, *DBP* diastolic blood pressure, *EF* ejection fraction, *LVEDd* left ventricular end diastolic diameter, *IVSd* interventricular septum thickness at diastole, *Pwd* posterior wall thickness at diastole, *LA* left atrium, *RVd* right ventricular basal diameter at apical 4 chamber view, *TV* tricuspid valve, *MV* mitral vlve, *E*/*E*’ mitral valve E wave by pulsed wave Doppler/mitral valve E’ wave by annular pulsed tissue Doppler, *Hb* hemoglobin, *TSH* thyroid-stimulating hormone

Echocardiography assessment revealed only nine persons (1.3%) with structural cardiac abnormalities, two had bicuspid aortic valve with isolated aortic regurgitation (AR), one of them had mild AR, and the other had moderate AR. Two cases of mitral valve prolapse (MVP); one of them had trivial MR while the other had flail anterior leaflet due to ruptured chordae with severe MR. One patient had atrial septal defect 1.5 cm with mild pulmonary hypertension (ESPAP = 40 mmHg) and right-sided chambers dilatation (RA area = 26 cm^2^, RV basal transverse diameter at apical four-chamber view = 4.7 cm). One patient had dextrocardia (situs inversus totalis) without other cardiac abnormalities. One had pulmonary hypertension (ESPAP = 50 mmHg) and right-sided chambers dilation (RA area = 22 cm^2^, RV basal transverse diameter at apical 4 chamber view = 4.6 cm) without apparent underlying cardiac abnormalities. Two patients had LVH, both were hypertensive subjects.

We did not detect rheumatic valvular heart disease. Also, we did not find HCM cases.

When we revised persons with abnormal findings, six of them had clear clinical abnormal findings and/or abnormal ECG, as shown in Table 5.

**Table 5:** Baseline ECG and clinical data of the nine subjects with their abnormal echocardiographic findings

Echocardiographic abnormalities	No.	ECG findings	Clinical
ASD	1	RVH	Splitted fixed S2
Dextrocardia	1	Dextrocardia pattern	Lt sided liver, heart sounds and LV apex at Rt side of the chest
Mild MVP, mild MR	1	Normal	Normal exam
Flail MV, severe MR	1	Non-specific	Muffled S1, systolic apical murmur, dyspnea NYHA II
BAV, Mild AR	1	Normal	Normal exam
BAV, moderate AR	1	Normal	AR murmur
LVH	2	LVH	Elevated BP
Pulmonary hypertension	1	Normal	Normal exam

*ASD* intra-atrial septal defect, *MVP* mitral valve prolapse, *MR* mitral regurgitation, *BAV* bicuspid aortic valve, *AR* aortic regurgitation, *LVH* left ventricular hypertrophy, *RVH* right ventricular hypertrophy, *Lt* left, *Rt* right, *NYHA* New York Heart Association, *BP* blood pressure

The money cost of echocardiography for those 795 persons was 198,750 Egyptian pounds (LE); their transportation cost was about 19.800LE. The total group time cost of the echocardiographic tests was 265 h, total time lost at echocardiography waiting room was 1590 h, total transportation time lose was 238 h, so total time cost was about 4000 h.

**Fig. 1:**
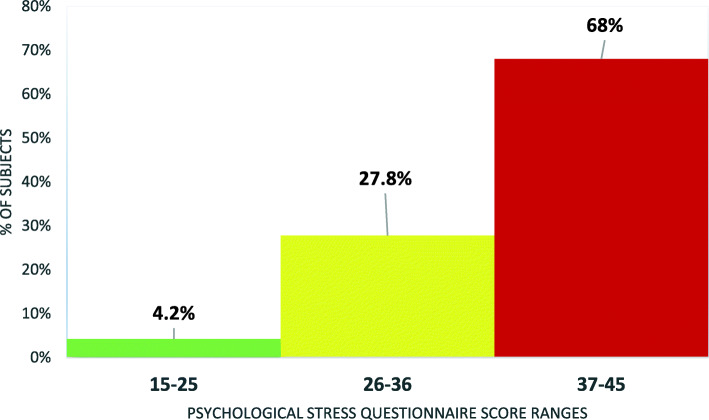
Distribution of studied subjects as regard psychological stress questionnaire score range

Psychological stress was another cost that they had to suffer from; every one of them was in a different degree of stress and fear of losing the job if there was an abnormal finding in the test. By using the psychological stress questionnaire, 33 participants (4.2%) had a range of scores between (15–25), which suggested a low sense of psychological stress due to echocardiographic examination, while 221 persons (27.8%) had a range of scores between (26–36), which suggested a moderate feeling of psychological stress due to the medical examination, and 541 subjects (68%) had a range of scores obtained between (37–45), which suggested a high sense of psychological stress because of a medical examination (Fig. 1).

## Discussion

As we noticed, the rising trend in many companies and even in governmental companies to use echocardiography as a routine assessment tool before employment for all subjects irrespective to medical history or clinical examination for fear of employing an apparently healthy person undiscovered cardiovascular disease which will lead to higher medical insurance and financial costs, beside the risk on the employee and his colleagues or general population. So we designed this study to judge this practice in the light of our country with its limited resources. Also, the previous studies studied different population age and was done in western countries with better health care system and lower incidence of rheumatic heart disease. And as we expect that the results will support our recommendations to the authorities to stop this unnecessary practice to save time and cost and to avoid unnecessary psychological stress.

In our study, the fourteen elements questionnaire only revealed 13 persons with abnormal findings and this can be attributed to the socioeconomic factor with the fear of losing the job which is so valuable for them so they tended to deny the symptoms and their personal and family history of cardiovascular diseases despite their previous orientation by physicians as regards the safety purpose of these data.

As regard time cost, the total time consumed was about 4000 h (≈166.7 day) which reflected as a significant delay on the services offered by echocardiographic unite for regular work.

And the total money spent for the examinations was 218550 Egyptian pounds which is considered a large burden in a developing country with subjects of low economic level, and in comparison with the few data added by echocardiography in the study, it looks unpractical and costly as a routine examination tool for detection of cardiovascular abnormalities in subjects without symptoms, clinical, and/or ECG abnormalities.

The study suggested that the participants suffered from various degrees of psychological stress caused by referral for echocardiography where 68% of them had severe psychological stress while 32% had mild to moderate psychological stress, which should be put into consideration while recommending this test.

From the 795 subjects who were routinely examined by echocardiography as a pre-employment test, only 9 subjects had abnormal findings; 6 of them already had abnormal clinical and/or ECG findings, so only three subjects (0.377%) had cardiac abnormalities that were detected only by echocardiography but their abnormalities were non-serious or non-significant forms (Table 5).

Lindekleiv H et al.’s study found that echocardiographic screening the general population for structural heart disease had no benefit for death or the risk of myocardial infarction and CV stroke. The prevalence of structural heart disease was 7.6%. They had a higher prevalence than our study due to the higher age of their studied group (mean age was 60 years) and their study contained a larger sample volume of 3272 subjects [14].

We found that 18% of the subjects had a heart rate be between 100 and 120 b/m (sinus tachycardia), which may reflect the increased psychological burden for fear of the rejection after the echocardiographic examination.

Of the seven subjects with elevated BP during screening, two were known to be hypertensive patients on medical treatment but not well controlled with evidence of LVH by ECG, and echocardiography confirmed the diagnosis of LVH in these two patients. Two of the remaining five subjects truly have grade 1 hypertension and were managed with lifestyle modifications, and their ECG and echocardiography were normal. The remaining 3 subjects had stress-induced elevated BP with normal home BP readings. In this group, echocardiography did not add new data.

Subjects with DM, family history of SCD, and family history of premature CAD were asymptomatic with normal cardiac examinations, ECGs, and echocardiography.

The two subjects with a history of single attack of syncope were diagnosed as neurogenic syncope (one of them syncopal attack occurred during hard work in hot weather and the other during prolonged fasting when he was in long-standing in a queue) without any other clinical, ECG or echocardiographic abnormalities.

As regards the 14 subjects with abnormal ECGs, five of them had abnormal echocardiographic findings (Table 4) while the others had normal echocardiography.

Despite the majority of examined subjects were from rural areas, we did not find rheumatic heart disease cases which were unexpected to the authors, but we tried to explain this by the wide empirical use of long-acting penicillin injection at a low threshold in children with symptoms mimic rheumatic fever or even just elevation in ESR or ASOT in the last few decades, but this number of subjects couldn't be enough to give such result, we think this point needs further screening with a much higher number of participants.

Also, hypertrophic cardiomyopathy (HCM) is the most common inherited heart disease; we did not find HCM cases and we attribute this to the relatively small study shamble for such disease with prevalence rates between 0.2 and 0.3%.

Data coming from the 25-year Italian experience for screening to detect HCM in young competitive athletes stated that although echocardiography is the main diagnostic test for HCM diagnosis, it is expensive and impractical as a screening test for large populations [2] and they recommended twelve-lead ECG (in addition to history and physical examination) as an alternative, cost-effective method for population-based screening and stated that 12-lead ECG may be as sensitive as echocardiography in detecting HCM in the young athletic population. They put in their consideration the significant socio-economic impact and need for the cultural background [15].

Because of the low prevalence of structural heart disease among the general population, echocardiography has traditionally not been considered justified in low-risk individuals, although it is recommended in screening asymptomatic individuals with a family history of sudden death or hereditary diseases affecting the heart or the great vessels [16].

### Limitations

Unequal gender distribution, limited age group, intra-observer variability, and the pretest bias of the examiners that young apparently healthy subjects will have normal examinations.

## Conclusion

The use of echocardiography as a routine pre-employment test added few data in patients without significant symptoms, ECG, and/or clinical findings, and on the other hand, it had significant economic and psychologic stress burden with unnecessary time consumption so we recommend against routine echocardiography for cardiac assessment in pre-employment assessment and to do it only for persons with symptoms suggestive of cardiac problems, abnormal clinical or ECG findings.

## Data Availability

The datasets used and/or analyzed during the current study are available from the corresponding author on reasonable request.

## Abbreviations

BP: Blood pressure
DM: Diabetes mellitus
SCD: Sudden cardiac death
IHD: Ischemic heart disease
SBP: Systolic blood pressure
DBP: Diastolic blood pressure
EF: Ejection fraction
LVEDd: Left ventricular end-diastolic diameter
IVSd: Interventricular septum thickness at diastole
Pwd: Posterior wall thickness at diastole
LA: Left atrium
RVd: Right ventricular diameter by mode
TV: Tricuspid valve
MV: Mitral valve
E/E': Mitral valve E wave by pulsed-wave Doppler/mitral valve E' wave by annular pulsed tissue Doppler
Hb: Hemoglobin
TSH: Thyroid-stimulating hormone
ASD: Intra-atrial septal defect
MVP: Mitral valve prolapse
MR: Mitral regurgitation
BAV: Bicuspid aortic valve
AR: Aortic regurgitation
LVH: Left ventricular hypertrophy
RVH: Right ventricular hypertrophy
Lt: Left
Rt: Right
NYHA: New York Heart Association
RA: Right atrium
RV: Right ventricle
ESPAP: Estimated systolic pulmonary artery pressure
RBBB: Right bundle branch block
LVOT: Left ventricular outflow tract
HCM: Hypertrophic cardiomyopathy
